# Left Atrial Appendage Transcatheter Occlusion with AMPLATZER™ Amulet™ Device: Real Life Data with Mid-Term Follow-Up Results

**DOI:** 10.5935/abc.20190138

**Published:** 2019-10

**Authors:** Mehmet Levent Şahiner, Ergun Baris Kaya, Cem Çöteli, Kudret Aytemir

**Affiliations:** Hacettepe Universitesi Tip Fakultesi - Department of Cardiogly, Ankara - Turkey

**Keywords:** Atrial Fibrillation, Atrial Appendage, Mortality, Echocardiography/methods, Cardiac Catheterization, Anticoagulants/therapeutic use

## Abstract

**Background:**

Left atrial appendage (LAA) occlusion is an alternative therapy for atrial fibrillation patients who have high embolic risk and contraindications to anticoagulant therapy.

**Objective:**

To evaluate the feasibility, safety, and mid-term outcomes of percutaneous LAA occlusion, including device-related thrombosis.

**Methods:**

Sixty consecutive patients who had undergone percutaneous LAA occlusion with AMPLATZER™ Amulet™ device from September 2015 to March 2018 were enrolled. Patients were followed for 21 ± 15 months (median - 20 months, interquartile range - 9 to 27 months). The postprocedural assessment was done at the 1^st^, 6^th^, and 12^th^ month. Patients were clinically evaluated, and transesophageal echocardiography was performed at each visit. We evaluated the condition of normality of variables using the Kolmogorov-Smirnov test. P-values < 0.05 were statistically significant.

**Results:**

The most common indication for the procedure was major bleeding with anticoagulants (n: 53, 88.3%). The procedure was completed successfully in 59 (98.3%) patients. Periprocedural mortality was observed in one patient. Postprocedural antiplatelet treatment was planned as dual or single antiplatelet therapy or low-dose anticoagulant therapy in 52 (88.1%), 2 (3.4%), and 5 (8.5%) patients, respectively. We found no clinically significant cerebrovascular events, device-related thrombus, or embolization in any patient during the follow-up. Two (3.4 %) patients presented significant peri-device leak (>3 mm) at the 1^st^ month evaluation, which disappeared at the 12^th^ month follow-up.

**Conclusion:**

We concluded that LAA occlusion using the Amulet™ LAA occluder can be performed with high procedural success and acceptable outcomes.

## Introduction

Atrial fibrillation (AF) is the most common type of sustained cardiac arrhythmia, especially in older adults.^1^ AF is associated with increased all-cause mortality and morbidity. The most significant AF morbidity is thromboembolic cerebrovascular events (CVEs). CVEs result in decreased quality of life and increased health care costs.^2^ Oral anticoagulants (OACs) are effective therapeutic options to prevent thromboembolic events.^2^ Randomized controlled studies and real-life studies showed that OAC drugs raise the risk of bleeding.^3^ The major bleeding risk with vitamin K antagonists and direct OACs should not be ignored, especially in patients with high bleeding risk.^4^-^6^ The balance between the protection from thromboembolic events and bleeding risk may be overbalanced towards bleeding. In this scenario, left atrial appendage (LAA) occlusion should be an alternative therapeutic option for some specific patient groups.^2^

According to current AF guidelines from the American College of Cardiology/American Heart Association (ACC/AHA) and the European Society of Cardiology (ESC), LAA occlusion (surgical or percutaneous) may be considered for stroke prevention in patients with AF and contraindications to long-term anticoagulant treatment with Class IIb and Level B recommendation.^2^,^7^

Due to the lack of large randomized controlled trials on LAA occlusion with the Amulet device, there are some gaps on clinical approaches for perioperative preparation, appropriate treatments for possible complications, and postoperative follow-up, including post-implant antithrombotic therapy. In this retrospective observational study, we aimed to emphasize challenges to LAA occlusion, evaluate possible perioperative complications, and how to deal with them. In addition, we intended to reveal real-life mid-term outcomes in our patient group and share our postprocedural antiplatelet regimen as an alternative option for patients who have very high stroke risk despite the LAA occlusion.

## Methods

### Population

Patients who had a major bleeding event with anticoagulant treatment, recurrent minor bleeding with at least two diferente anticoagulant treatments, or any life-threatening bleeding risk, such as high risk of falling or idiopathic thrombocytopenic purpura, were evaluated for suitability for LAA occlusion. Individuals who had less than one-year survival, were under critical non-cardiac status, and did not accept any interventional procedure were excluded. This retrospective observational study included 60 patients who had undergone percutaneous LAA occlusion with the Amulet device in the Hacettepe University Hospital Cardiology Clinic between September 2015 and March 2018. All patients signed the informed consent form, and the local ethics committee approved the procedures.

Symptomatic bleeding in a critical area or organ (intracranial, intraspinal, retroperitoneal etc.) and bleeding causing a fall in hemoglobin level of 20 g/L or more or leading to transfusion of two or more units of whole blood or red cells were considered a major bleeding, in accordance with International Society on Thrombosis and Haemostasis (ISTH) recommendations.^8^

### Statistical analysis

We performed the statistical analysis using the SPSS statistical software (version 20; SPSS Inc., Chicago, IL, USA). Descriptive and categorical variables were presented as frequencies and percentages. Continuous data with normal distribution were expressed as means ± SD. Quantitative variables without normal distribution were described as median and interquartile range. We evaluated the condition of normality using the Kolmogorov-Smirnov test. The Student’s t-test or Mann-Whitney test compared the numerical variables, as appropriate. P-values < 0.05 were statistically significant.

### The AMPLATZER™ Amulet™ Left Atrial Appendage Occluder

The AMPLATZER™ Amulet™ Left Atrial Appendage Occluder (ST Jude Medical, Minneapolis, Minnesota) was used in all 60 patients for LAA occlusion. The AMPLATZER™ Amulet™ device is a self-expanding nitinol device with two parts (a lobe and a disc) pre-assembled on a single cable. Depending on the size of the device, a 12 to 14 French delivery catheter is used.

### Measurement of left atrial appendage dimensions

Multidetector Computed Tomography (MDCT) was performed in 31 patients who had normal kidney function to evaluate the LAA anatomy. The LAA landing zone was measured with the MDCT in these 31 patients. All patients underwent transesophageal echocardiography (TEE) to guide the device selection and evaluate cardiac function. The device size was selected by using 3D TEE and MDCT when available. All patients had the size of LAA ostium and the device landing zone measured by TEE. TEE results were compared with MDCT ones in patients who had preprocedural MDCT. The relationship between LAA and pulmonary artery was evaluated in patients who had preprocedural MDCT.

### Left atrial appendage occlusion procedure

As a routine preprocedural approach, standard transthoracic echocardiogram and TEE were performed before LAA occlusion in all patients to evaluate the shape and size of LAA and to reveal the presence of thrombus in LAA. TEE was performed after intravenous fluid infusion to avoid undersizing of LAA due to hypovolemia. The intravenous fluid infusion volume was determined according to the patients’ physical examination, B-type natriuretic peptide (BNP) levels, and left ventricular ejection fraction. Left atrial pressure was also measured to determine the optimal intravascular volume status during the procedure. Patients with normal renal function underwent multislice cardiac computed tomography for optimal evaluation of LAA anatomy, size, and the relationship between LAA and related cardiovascular structures.

All patients undergoing percutaneous LAA occlusion procedure were under general anesthesia and intubated for better TEE guidance. Transseptal puncture was conducted with fluoroscopy and 3D TEE guidance at the inferoposterior site of the interatrial septum when the patient had no anatomic variations that could prevent optimum orientation. After a successful transseptal puncture, the delivery catheter was placed in the left atrium. The Amulet™ LAA occlusion device was then advanced to some extent out of the delivery sheath, the lobe of the device formed a ball shape, and the delivery sheath was placed in the LAA with a counterclockwise rotation. After confirming the optimum settlement in the LAA with TEE, the lobe of the device was opened with further advancement. After the proper placement of the lobe in the LAA, the disc was opened at the LAA ostium with the withdrawal of the delivery sheath. Relationships between the occlusion device and the circumflex artery and mitral valve were checked with 3D TEE, and radiopaque contrast was injected in the delivery sheath to evaluate para-device leak. Before being released, the device was pulled back with acceptable strength to check the stability. After all these steps, the occlusion device was released, and the relationships between the device and LAA, circumflex artery, and mitral valve were evaluated by 3D TEE. Periprocedural anticoagulation was maintained by IV heparin infusion with activated clotting time (ACT) control.

### Postprocedural antiplatelet therapy

Postprocedural antiplatelet therapy was planned as dual or single antiplatelet therapy or low-dose anticoagulant therapy. This individualized therapy was designed according to patients’ thromboembolism as well as bleeding risk.

### Postprocedural follow-up

The patients were reevaluated with transthoracic echocardiography at the 1^st^, 6^th^, and 12^th^ month. TEE was performed at all three visits. The patients were evaluated clinically and with TEE annually after the first year post-procedure.

## Results

### Baseline characteristics

This study involved 60 patients (mean age wasn72.3 ± 20.1 years) who had undergone percutaneous LAA occlusion with the AMPLATZER Amulet device in the Hacettepe University Cardiology Clinic between September 2015 and March 2018. The sample consisted of 35 women (58.3%) and 25 men (41.7%). Table 1 lists all baseline characteristics.

**Table 1:** Baseline Characteristics and LAA Occlusion Indications

Baseline Characteristics	n = 60
Mean Age	72.3 years ± 20.1 years
Female Gender, n (%)	35 (58.3%)
Hypertension, n (%)	56 (93.3%)
Diabetes Mellitus, n (%)	22 (36.6%)
Heart Failure, n (%)	23 (38.3%)
Cerebrovascular Event, n (%)	17 (28.3%)
Ischemic, n (%)	13 (21.6%)
Hemorrhagic, n (%)	3 (5.0%)
Ischemic and hemorrhagic, n (%)	1 (1.6%)
Chronic Kidney Disease, n (%)	29 (48.3%)
Stage 3 (GFR: 30% ≤ 59%)	14 (23.3%)
Stage 4 (GFR: 15% ≤ 29%)	7 (11.6%)
Stage 5 (GFR: ≤ 14%)	8 (13.3%)
Atherosclerotic Heart Disease, n (%)	40 (66.7%)
Peripheric Artery Disease, n (%)	7 (11.6%)
**Atrial Fibrillation**	
Paroxysmal, n (%)	13 (21.6%)
Persistent, n (%)	47 (78.3%)
**Preprocedural Anticoagulation**	
Yes, n (%)	57 (95.0%)
Rivaroxaban, n (%)	30 (50.0%)
Warfarin, n (%)	4 (6.6%)
Dabigatran, n (%)	10 (16.7%)
Apixaban, n (%)	13 (21.7%)
**Follow Up, mean months ± SD, (median months, 1^st^ and 3^rd^ quartile)**	21 ± 15 months (20 months, interquartile range of 9 to 27 months)
**Left Atrial Appendage Occlusion Indications**	
Major bleeding, n (%)	53 (88.3%)
Gastrointestinal, n (%)	36 (60.0%)
Hemoptysis, n (%)	11 (18.3%)
Hemorrhagic SVE, n (%)	4 (6.6%)
Pericardial, n (%)	1 (1.7%)
Retroperitoneal, n (%)	1 (1.7%)
Chronic Kidney Disease and Labile INR, n (%)	4 (6.6%)
Idiopathic Thrombocytopenic Purpura, n (%)	1 (1.7%)
Cerebral Angiopathy, n (%)	1 (1.7%)
Bronchiectasis, n (%)	1 (1.7%)
**Thromboembolic and Bleeding Risk Scores**	
CHADS_2_ mean ± SD	2.75 ± 2.25
CHA_2_DS_2_-VASc mean ± SD	4.61 ± 2.61
HASBLED mean ± SD	4.32 ± 3.32
ORBIT mean ± SD	4.8 ± 2.8

The most common reason for LAA occlusion was major bleeding with OAC treatment (n: 53, 88.3%). The most common type of major bleeding was gastrointestinal bleeding (n:26, 57,8%).

Fifty-seven patients took OACs before LAA occlusion. The most common preprocedural anticoagulant used was rivaroxaban, in 30 (50.0%) patients. Warfarin, dabigatran, and apixaban were used by 4 (6.6%), 10 (16.7%), and 13 (21.7%) patients, respectively.

### Thromboembolic events and bleeding risk scores

CHADS2, CHA2DS2-VASc, HAS-BLED, and ORBIT bleeding scores were calculated for all patients, and the average values of these scores were 2.75 ± 2.25, 4.61 ± 2.61, 4.32 ± 3.32, and 4.8 ± 2.8, respectively. Table 1 lists bleeding and thromboembolic event risk scores separately.

### Device dimensions

The smallest implanted device had 16 mm and the biggest, 31 mm. Devices of 20 mm were implanted in 11 patients and of 25 mm in 16 patients.

### Procedural Outcomes

The LAA occlusion device was implanted successfully in all 60 patients. No patient showed device embolization. One patient presented postprocedural major complication and mortality. Fifty-nine patients were discharged without any disabling complication. Six patients had periprocedural bleeding. All of them were associated with an access point, and only one of these patients needed a postprocedural blood transfusion. Periprocedural stroke, transient ischemic attack (TIA), and systemic embolization were not observed in any patient during hospital follow-up. The mean postprocedural hospital length of stay was 1.33 days (median of 2 days, interquartile range of 1 to 3 days).

### Periprocedural complications

The percutaneous LAA transcatheter occlusion device was implanted successfully in all 60 patients. However, one patient presented a postprocedural major complication. This patient had been referred to emergency surgery due to pulmonary artery rupture. Despite the surgical repair of the pulmonary artery injury, the patient did not survive.

Two patients had postprocedural pericardial effusion, both self-limited and not requiring pericardiocentesis. Two patients started postprocedural ibuprofen and colchicine therapy.

**Figure 1:**
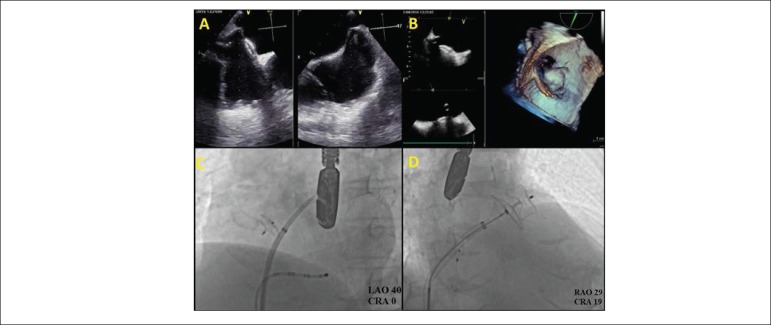
a) Transesophageal echocardiography image of the transseptal puncture needle tenting the inferoposterior site of the interatrial septum and atrial septal defect closure device, b) Tridimensional transesophageal echocardiography guidewire image after the transseptal puncture, c) Fluoroscopic image of the transseptal puncture on the inferoposterior site of the interatrial septum and atrial septal defect closure device, d) Fluoroscopic image of the device before being released.

Some clinical and anatomic patient features created problems for the procedural approach, but none of them prevented successful implantation. Five patients had a thrombus formation at the bottom of the LAA. The thrombus was attached to the LAA occlusion device in these patients. One patient had an atrial septal defect (ASD) closure device at the interatrial septum, which had been previously implanted. Generally, an ASD closure device in place is considered challenging for the transseptal puncture, but this patient had the transseptal puncture performed at the inferoposterior side of the interatrial septum, which is the most suitable puncture

location for LAA occlusion. The LAA occlusion procedure was performed successfully in this patient without any damage to the ASD closure device (Figure 1).

### Patients’ postprocedural antiplatelet therapy

**Figure 2:**
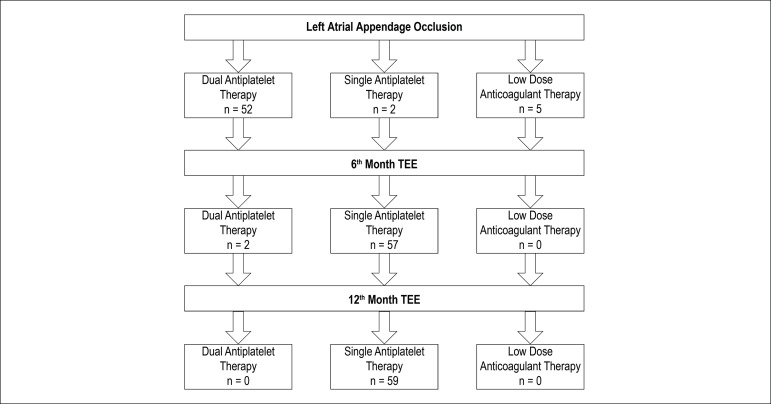
Postprocedural antithrombotic treatment.

In most patients, postprocedural antiplatelet therapy consisted of acetylsalicylic acid (100 mg qd) and clopidogrel (75 mg qd). Dual antiplatelet therapy (DAPT) continued for 6 months after the procedure in 53 patients. DAPT was modified to the single antiplatelet therapy (acetylsalicylic acid or clopidogrel) if the absence of thrombus formation over the device was confirmed and peri-device leak was not found. Fifty-three patients under DAPT showed no device-related thrombus (DRT) or peri-device leak at the 6th month follow-up TEE. Accordingly, these patients continued with single antiplatelet therapy thereafter. Two patients under DAPT after the procedure had TIA during follow-up, which extended the DAPT for 12 months. Single antiplatelet therapy with acetylsalicylic acid was considered in only two patients due to the high bleeding risk. They continued to use single antiplatelet during their entire follow-up. Five patients used a low-dose OAC agent after the procedure. Four of them used apixaban 2.5 mg BID, and one took dabigatran 110mg BID until their 6th month evaluation. None of them had thrombus over the device or peri-device leak at the 6th month TEE. Thus, they continued their antithrombotic treatment with single antiplatelet therapy after their 6th month visit (Figure 2).

### Follow-up outcomes

The patients were evaluated at the 1st, 6th, and 12th month after discharge, undergoing annual assessments afterward. Two patients died during the follow-up. Median follow-up duration was 20 months (interquartile range of 9 to 27 months). The first patient died due to decompensated heart failure six months after the LAA occlusion. The second patient died from a non-cardiac condition.

Clinically manifested stroke did not occur during the follow-up period. Two patients presented TIA-like symptoms and underwent cerebrovascular scanning and TEE. The exams showed no significant findings. These patients had a neurology consultation, and TIA was considered. Their DAPT was extended to 12 months. Both patients were discharged without neurological deficit, and brain imaging showed no evidence of new ischemic lesions. Besides these two cases, the most important thromboembolic clinical event was pulmonary embolism in one patient two months after LAA occlusion.

Four patients had bleeding. One required hospitalization and blood transfusion three months after the procedure. This patient had melena. Gastrointestinal bleeding was confirmed with endoscopy and colonoscopy. DAPT was switched to single clopidogrel therapy, and the patient was discharged five days after hospitalization. The bleeding was not significant in the other three patients, and they did not require hospitalization or blood transfusion. Two of them had epistaxis, and one had epidermal petechiae.

**Figure 3:**
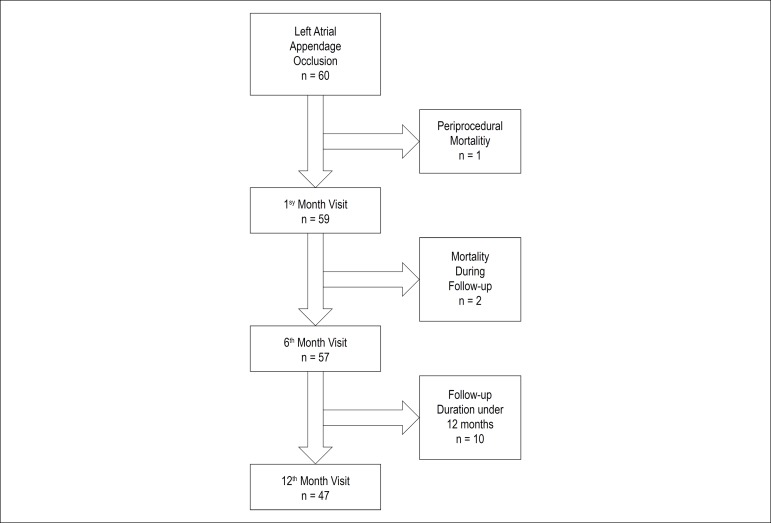
Patients’ follow-up.

Routine TEE was performed at the end of the 1st, 6th, and 12th month after the procedure. In 10 patients, the follow up-duration was shorter than 12 months, and they were evaluated only at the 1st and 6th month (Figure 3). No patient showed device thrombus or embolization at any visit. Two (3.3%) patients had significant peri-device leak (>3 mm) at the 1st and 6th month visit. These two patients did not present peri-device leak at the 12th month TEE (Table 2).

**Table 2:** Procedural and Follow-up Outcomes

Procedural Outcomes	Patients (n = 60)
Technical Success	60 (100%)
Procedural Success	59 (98.3%)
Periprocedural Mortality	1 (1.6%)
Periprocedural Morbidity	9 (15.0%)
Major Bleeding	0 (0.0%)
Minor Bleeding	6 (10.0%)
Stroke	0 (0%)
Systemic Embolization	0 (0%)
Device Embolization	0 (0%)
Pericardial Effusion	2 (3.2%)
Pericardial Tamponade	1 (1.6%)
**Follow-up Outcomes**	**Patients (n = 59)**
Mortality	2 (3.4%)
Stroke/TIA	2 (3.4%)
Ischemic Stroke	0
Hemorrhagic Stroke	0
TIA	2 (3.4%)
Pulmonary Thromboembolism	1 (1.7%)
Life Threating or Major Bleeding	1 (1.7%)
Minor Bleeding	3 (5.1%)
**Major Findings in Follow up TEE**	
Peridevice Leak (>3 mm) at 1^st^ month	2 (3.4%)
Peridevice Leak (>3 mm) at 6^th^ month	0
Device Related Thrombus	0
Device Embolization	0

TIA: transient ischemic attack; TEE: transesophageal echocardiography.

## Discussion

We used the AMPLATZER™ Amulet™ LAA occluder for percutaneous LAA occlusion in a series of patients and reported mid-term data on its safety and efficacy.

The PROTECT-AF and PREVAIL studies provided large-scale randomized clinical trial evidence that LAA occlusion with the Watchman device could be non-inferior to anticoagulation for CVEs in patients with non-valvular AF.^9^,^10^ On the other hand, large-scale randomized data on other LAA occlusion devices are limited.

The AMPLATZER Amulet device is currently being evaluated in a randomized controlled trial (Amulet IDE Trial; ClinicalTrials. gov Identifier: NCT02879448) and long-term randomized trial data has not been published yet. Thus, real-life data, multicenter registries, and meta-analysis of these studies still give us important information about the efficacy and safety of LAA occlusion with the Amulet device.

In our series of 60 patients, the LAA occlusion procedure was successfully completed without major complications in 98.3% of cases. Our procedural success rate was similar to previous studies.^11^-^13^ Landmesser et al.^12^ reported that the procedural success of LAA occlusion with the Amulet device was 99.0% in their multicenter registry, which included 1,088 patients.^12^ They reported peri-device leak in 2% of patients during follow-up. In accordance with these data, we identified significant peri-device leak (>3 mm) in two patients (3.3%) with TEE at the 1st month after the procedure.^12^ However, they showed no significant peri-device leak at the 6th month. We considered that this result might be due to the continued endothelization process on the closure device until six months after the procedure.

We found no significant disabling CVEs during follow-up. Only two patients had TIA, but with no neurological sequelae, and their cranial imaging did not reveal significant ischemic lesions. On the other hand, previous multicenter registries showed that there is still a risk of thromboembolic events despite LAA occlusion. Regueiro et al.^14^ recently reported that 7 out of 101 patients (6.9%) had a stroke after LAA occlusion in 4.2 years of follow-up, 6 of them related to thromboembolic events.^14^ AMPLATZER cardiac plug constituted most of the devices used in this study (82%), while Amulet was used only in 3 patients. They discharged 70% of the patients under DAPT and those using a single antiplatelet agent. We used the Amulet device in all our patients and discharged 96% of the patients with DAPT, which continued up to 6 months after LAA occlusion. This fact might be one of the reasons for the low incidence of CVEs in the follow-up of our patient group. The relatively short follow-up duration and the smaller sample size in our study could also be reasons for this difference. 

DRT has been reported in 0-17% of patients after LAA occlusion.^15^ Recently, some concern has been raised that DRT formation after LAA occlusion may be more frequent than expected. Fauchier et al.^16^ reported that, among 469 patients who underwent LAA occlusion, the incidence of DRT was 7.2% in imaged patients during a mean follow-up of 13 ± 13 months.16 Thrombus over the device was an independent predictor of ischemic events. The Watchman device constituted most (58%) of devices used for LAA occlusion in this study, while the Amulet device was used in 97 patients. Interestingly, DAPT at discharge was associated with a lower risk of thrombus, and only 23.2% of the study group was discharged with this treatment. Costa et al.^17^ published patient outcomes over a 12-month follow-up and found no DRT.^17^ We did not observe DRT in our patients with TEE imaging at the 1st, 6th, and 12th month after LAA occlusion, corroborating their results.^17^ There is some controversy among studies regarding thrombus formation over the device. The individualized antiplatelet treatment may explain this difference in our patient series. We planned the antiplatelet regimen according to the patients’ risk of stroke and thrombus formation over the device. DAPT was administered to most patients (88.3%) at discharge in our group. In addition, we planned an extensive antiplatelet therapy for patients who had peri-device leak at follow-up. Also, five of our patients had thrombus in the LAA before the procedure. These patients underwent low-dose anticoagulant therapy for six months. As peri-device leak and presence of thrombus in the LAA before the procedure were considered risk factors for thrombus formation on the closure device, we decided to individualize the antiplatelet therapy of these patients. Moreover, the relatively small sample size may be another cause for this discrepancy.

**Figure 4:**
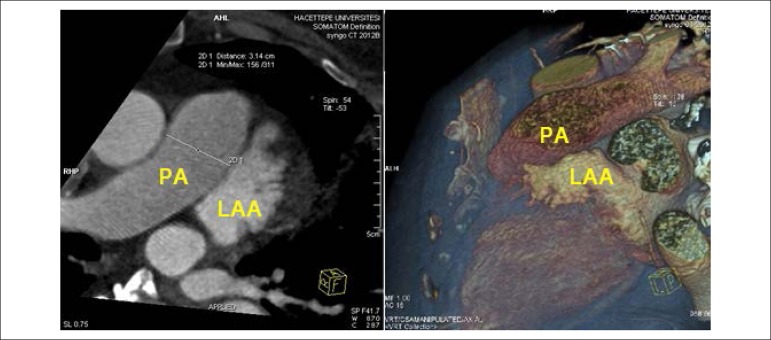
Computed tomography image of the neighborhood between pulmonary artery and left atrial appendage.

Our series had only one major periprocedural complication. The indication of LAA occlusion for this patient was preprocedural hemorrhagic CVE with an effective dosage of dabigatran. LAA occlusion was planned as thromboembolic prevention for this patient. Nonetheless, we observed a major periprocedural complication during the procedure. In this patient, the lobe hooks erupted from the LAA and damaged the pulmonary artery. These hooks are designed to allow better implantation and fixation of the device. When we reevaluated the preprocedural MDCT, we noted the close neighborhood between LAA and pulmonary artery. This close relationship resulted in pulmonary artery rupture. Although referred to urgent surgery, the patient did not survive. Previous case reports showed that postprocedural pulmonary artery rupture could be an early or delayed complication.^18^,^19^ Most of these case reports mentioned that this complication is related to the anatomical relationship between LAA landing zone and pulmonary artery.^18^,^19^ Halkin A. et al.^20^ classified this relationship according to the contact point between LAA and pulmonary artery and they emphasized that the type 2 (proximal contact) relationship has a higher pulmonary artery rupture risk than the others.20 We reevaluated the relationship between pulmonary artery and LAA in our patient after this study and found that it was a type 2 relationship (Figure 4).

Thrombus presence in LAA is considered a contraindication for LAA occlusion.^21^ In our series, we detected thrombus at the bottom of the LAA in five patients. We considered that the thrombus at distal LAA could be attached to the LAA occlusion device, with a modified technique and minimal manipulation of catheters in the left atrium. Consequently, the procedures were performed successfully with no periprocedural neurological complications. We have reported one of these cases previously.^22^ Tarantini et al.^23^ recently reported in their multicenter study that LAA occlusion could be safely and effectively performed in 28 patients with distal LAA thrombus.^23^ In line with these findings, we also suggest that LAA occlusion could be successfully conducted in patients with distal thrombus in experienced centers. However, the procedure should be canceled if the thrombus is located at the proximal LAA.

Percutaneous LAA occlusion is a complex procedure that has some periprocedural risks as we mentioned before. Consequently, preprocedural patient evaluation, patients with appropriate indications, and operator experience are very important to avoid possible complications.

In our study, we demonstrated that LAA occlusion using the Amulet™ LAA occluder could be performed with high procedural success. In our series, all but one of the procedures were completed safely without complications. We did not find any clinical events directly related to AF or the LAA procedure during postprocedural follow-up. On the other hand, further large-scale randomized trials and long-term outcome data are necessary to verify the efficacy and safety of LAA occlusion using the Amulet™ LAA occluder device.

### Limitations

This study was not designed as a randomized prospective controlled trial; consequently, it has some limitations. First, we did not have a control group to compare the effectiveness of LAA occlusion in preventing thromboembolic events. Second, our mean follow-up duration was relatively short, and long-term outcomes of LAA occlusion cannot be inferred from our results. However, the LAA occlusion procedure and Amulet device are relatively new, and data about this device are limited. Therefore, studies like ours are still important and valuable to show the performance of LAA occlusion with the Amulet device. Third, we performed LAA occlusion in different clinical scenarios, such as thrombus formation in the LAA. There are some studies about these challenging conditions and the safety of LAA occlusion. Our series had similar results to those found in the literature. Due to the lack of consensus on adjuvant antithrombotic therapeutic strategies, we individualized the antiplatelet therapy after the procedure. However, our study population was relatively small for us to recommend an antithrombotic regimen after the procedure. Large-scale studies are necessary to make such recommendations.

## Conclusion

LAA occlusion is an important and effective therapeutic option for selected AF patients with an increased risk of bleeding with anticoagulant treatment. Nevertheless, the procedure has some significant periprocedural risks, including death. Consequently, LAA occlusion should be performed in carefully selected patients with increased thromboembolic risk, who have at least one-year survival expectation and cannot tolerate OACs or had clinically important bleeding events.

Since LAA occlusion can be a challenging procedure, it should be performed by experienced operators with optimal skills and in collaboration with a heart team, including surgeons, neurologists, and experts on cardiovascular imaging.
